# Supplementary material for: IFN-gene signatures in B cells following influenza A and B virus infection and influenza vaccination
Source: EMBO Mol Med. 2026 Mar 9;18(4):1456–77. doi: 10.1038/s44321-026-00395-8 (PMC13083893; doi:10.1038/s44321-026-00395-8)
Supplement: Supplementary file 1 — Appendix [file 44321_2026_395_MOESM1_ESM.pdf]

IFN-gene signatures in B cells following influenza A and B virus infection and influenza vaccination

Wuji Zhang *et al.*

Appendix

	Page
**Appendix Figure S1.** Humoral responses of influenza patients and vaccinees.	2
**Appendix Figure S2.** scRNA sequencing reactions and quality control.	3
**Appendix Figure S3.** Violin plots of top DEGs for phenotype, isotype and activation markers.	4
**Appendix Figure S4.** Violin plots of top DEGs for B cell signalling and proliferation and atypical B cell markers.	5
**Appendix Figure S5.** HA-specific B cells expanded at VAX d28.	6
**Appendix Figure S6.** BCR gene segment usages in expanded HA-specific B cells and atypical B cells.	7
**Appendix Figure S7.** DEGs between vaccination and IAV infection.	8
**Appendix Figure S8.** Flow cytometry gating strategy.	9
**Appendix Figure S9.** Influenza virus infectivity in A549 cell lines.	10

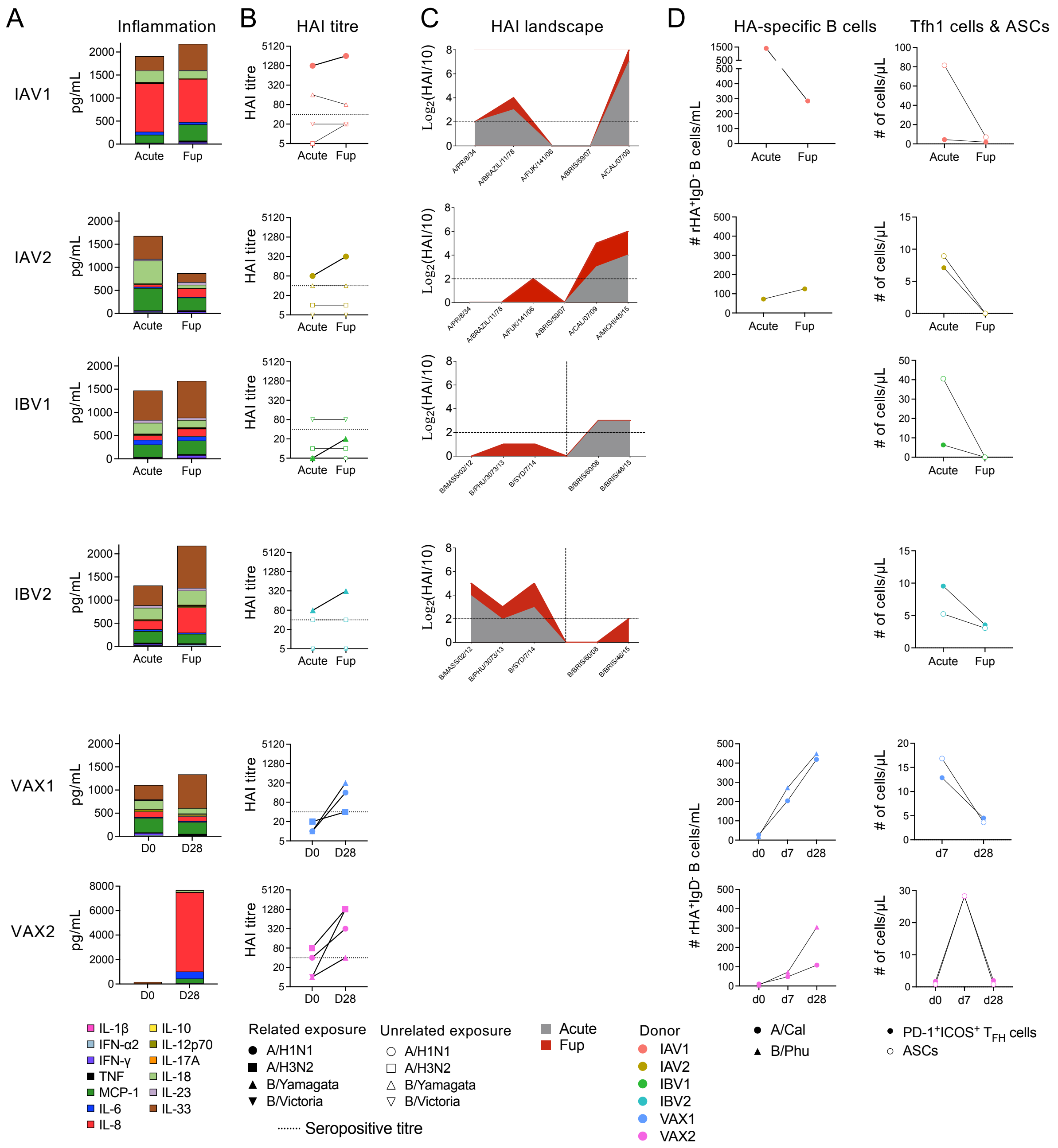

Appendix Figure S1. Humoral responses of influenza patients and vaccinees. (**A**) Cytokines, (**B**) HAI titre, (**C**) HAI landscape, and (**D**) HA-specific B cells, activated Tfh1 and ASC numbers of influenza patients and vaccinees.

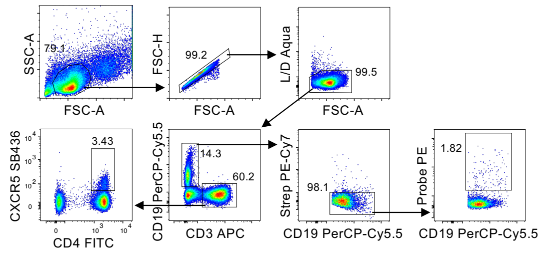

B

GEM reaction	Estimated Number of Cells	Mean Reads per Cell	Median Genes per Cell	Number of Reads	Valid Barcodes	Sequencing Saturation
GEM 1	9,230	81,235	1,303	749,805,079	83.00%	87.10%
GEM 2	6,152	124,379	1,420	765,184,606	84.20%	91.40%
GEM 3	8,389	89,920	1,344	754,343,107	83.30%	89.70%
GEM 4	8,731	83,553	1,313	729,503,334	82.90%	88.10%

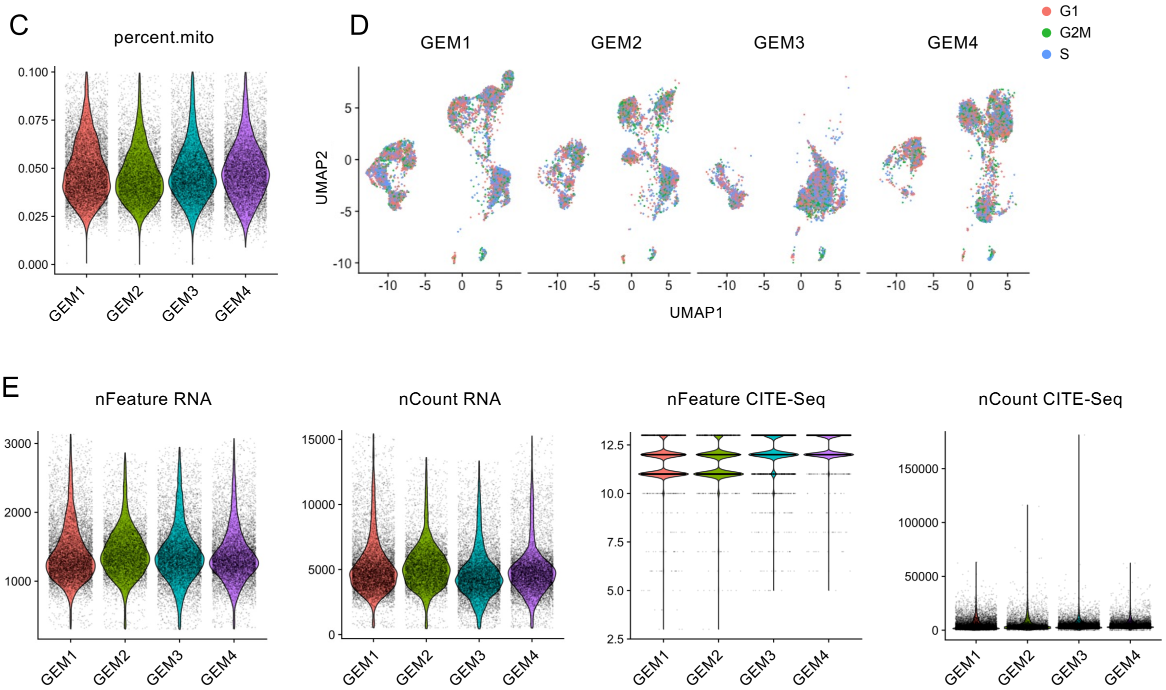

Appendix Figure S2. scRNA sequencing reactions and quality control. (**A**) Gating strategy of HA-specific B cell FACS-sorting for scRNA-seq. (**B**) Number of cells, reads, genes and percentage of valid barcodes and sequence saturation. (**C**) Proportion of mitochondrial content; (**D**) UMAP distribution of cell phase; and (**E**) number and counts of RNA and CITE-Seq features.

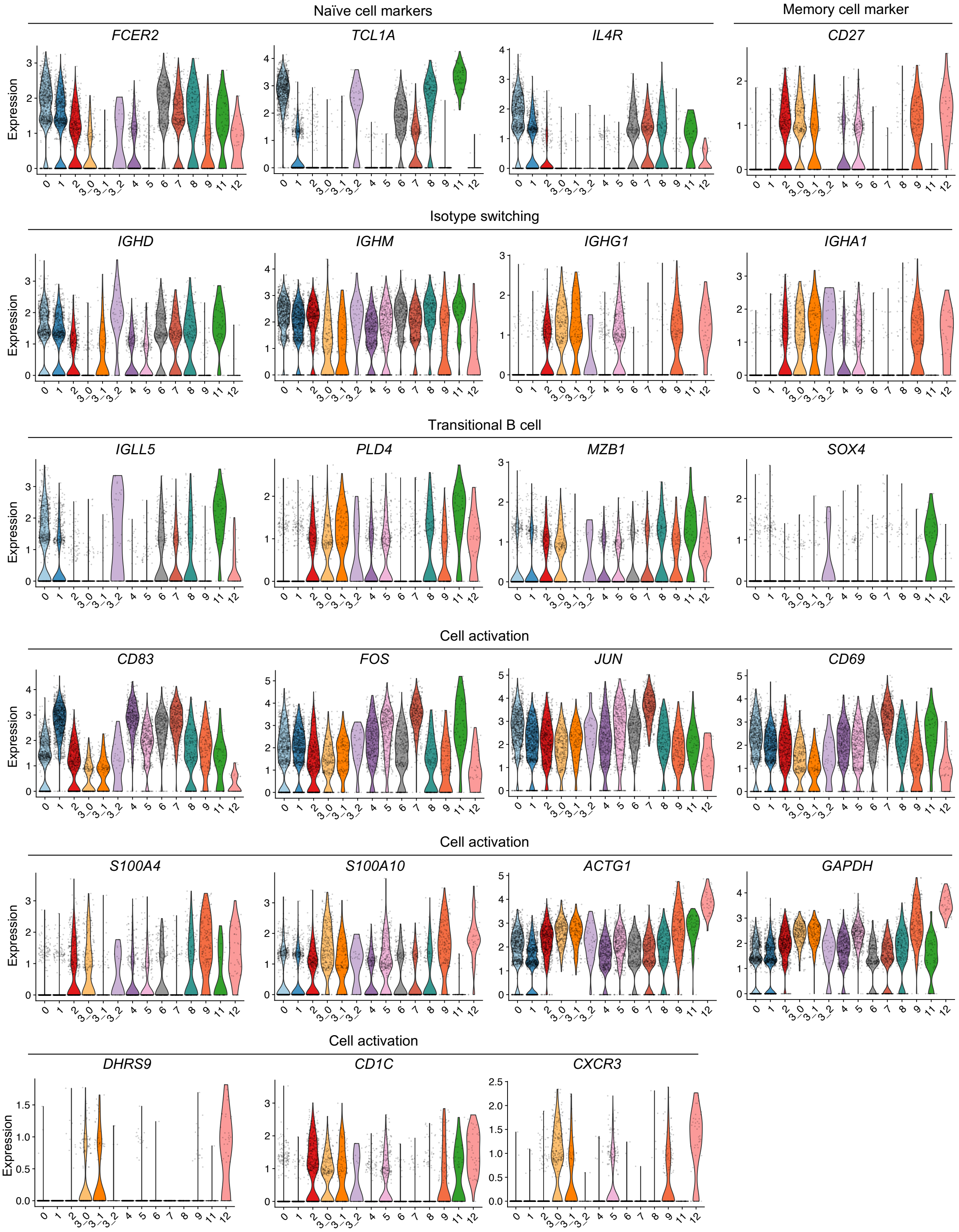

Appendix Figure S3. Violin plots of top DEGs for phenotype, isotype and activation markers. Violin plots displaying expression levels of DEGs shown in Fig. 1f associated with phenotypic and isotypic characterization and B cell activation for each B cell cluster.

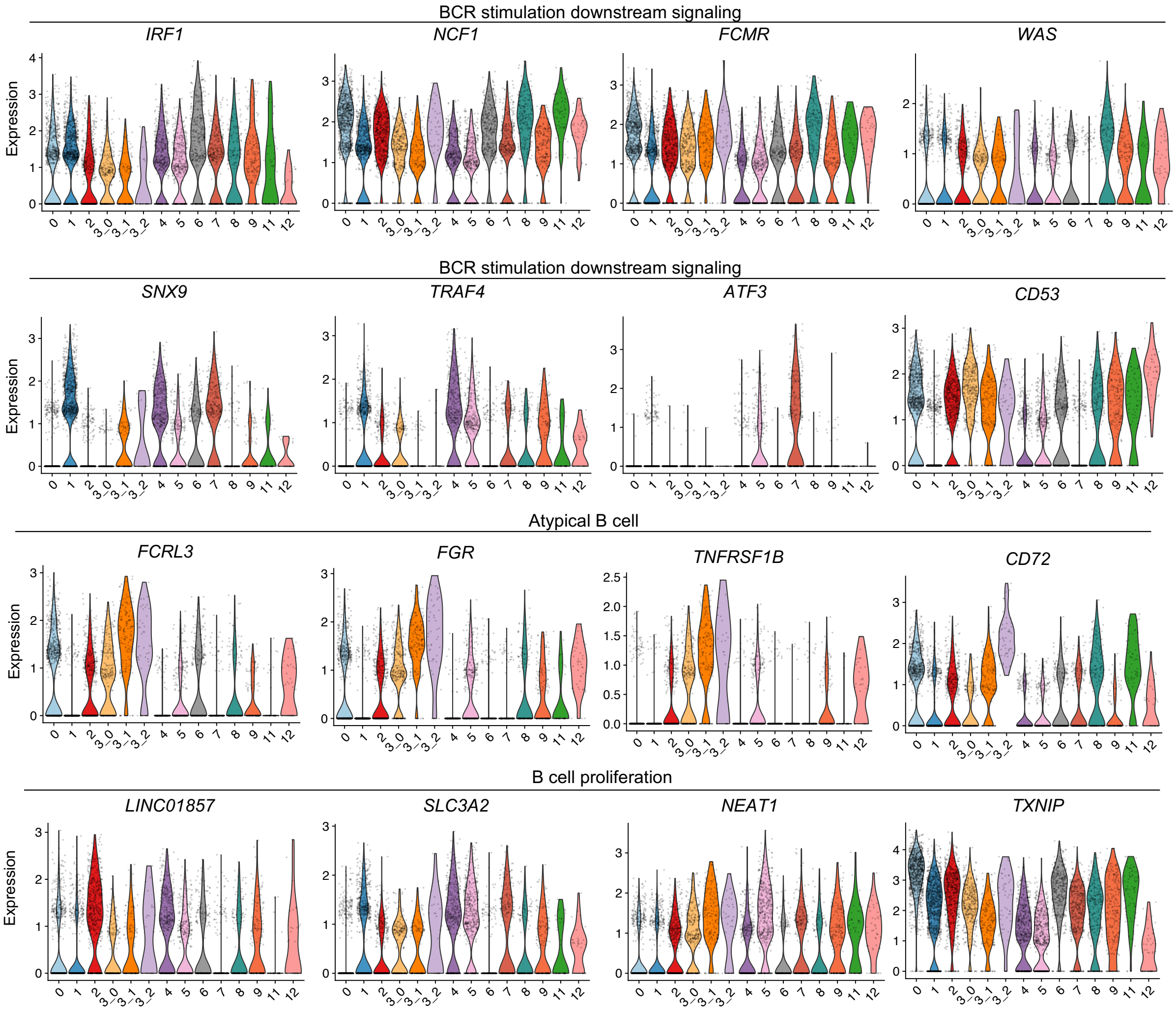

Appendix Figure S4. Violin plots of top DEGs for B cell signalling and proliferation and atypical B cell markers. Violin plots displaying expression levels of DEGs shown in Fig. 1f associated with B cell signalling and proliferation and atypical B cell markers for each B cell cluster.

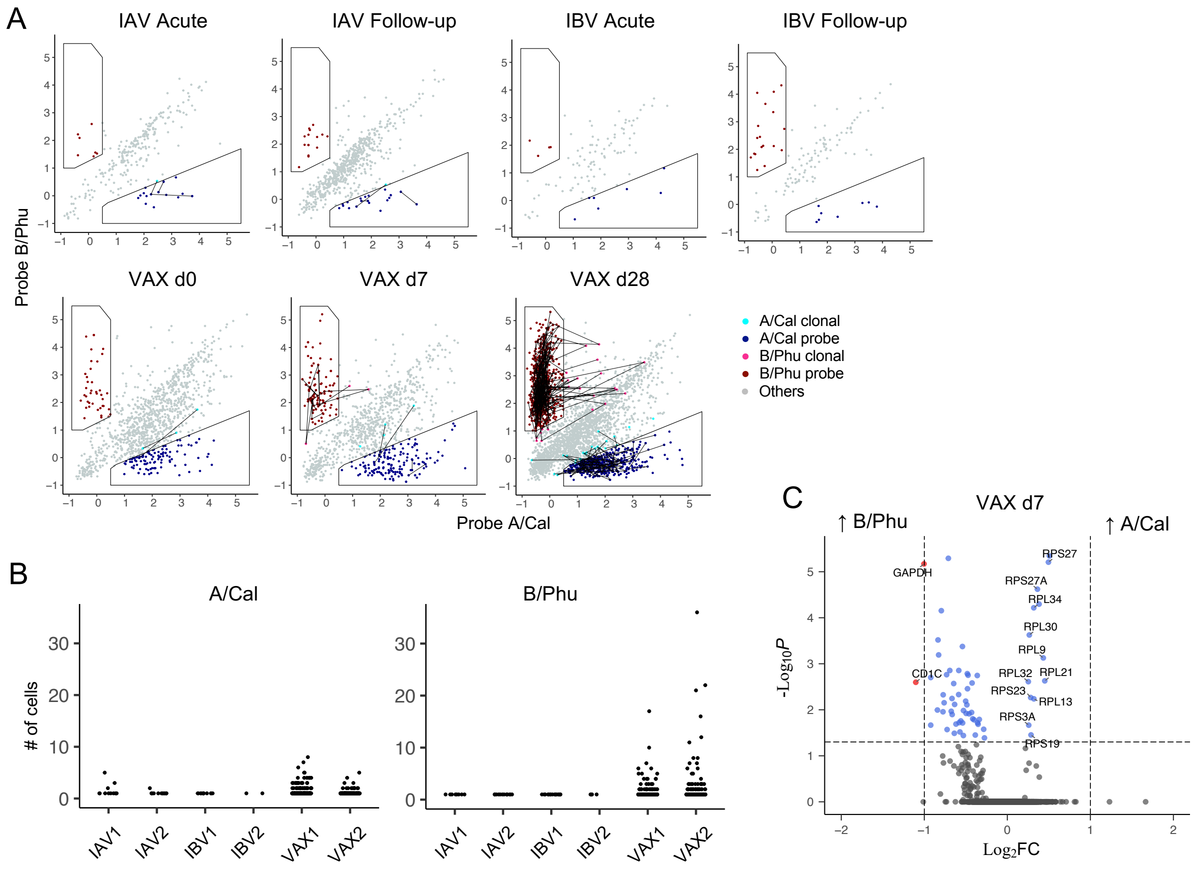

Appendix Figure S5. HA-specific B cells expanded at VAX d28. (**A**) HA-specific clonal B cells split by each infection/vaccination-time-point status. (**B**) Number of B cells in each B cell clone. Connected dots indicate HA probe-specific clonal B cells with shared BCR sequences (if any). (**C**) Volcano plot showing fold-changes of DEGs for A/California vs B/Phuket HA-specific B cells at VAX d7.

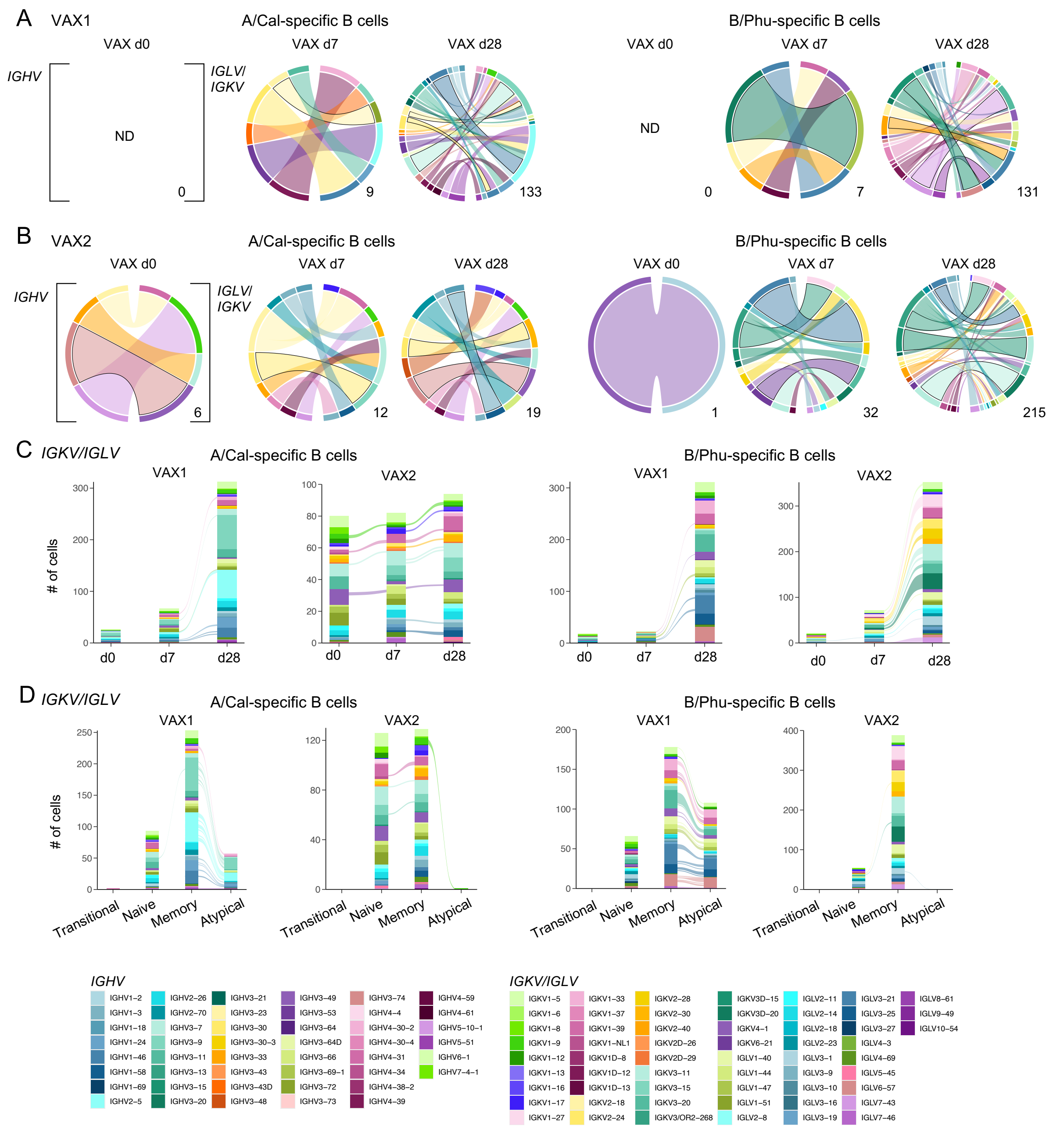

Appendix Figure S6. BCR gene segment usage in expanded HA-specific B cells and atypical B cells. (**A-B**) Circos plots of *IGHV* and *IGLV*/*IGKV* gene segment pairings of expanded HA-specific B cells for (**A**) VAX1 and (**B**) VAX2. (**C-D**) Alluvial plots of *IGKV/IGLV* gene segment usage for all HA-specific B cells across (**C**) timepoints or (**D**) B cell phenotypes.

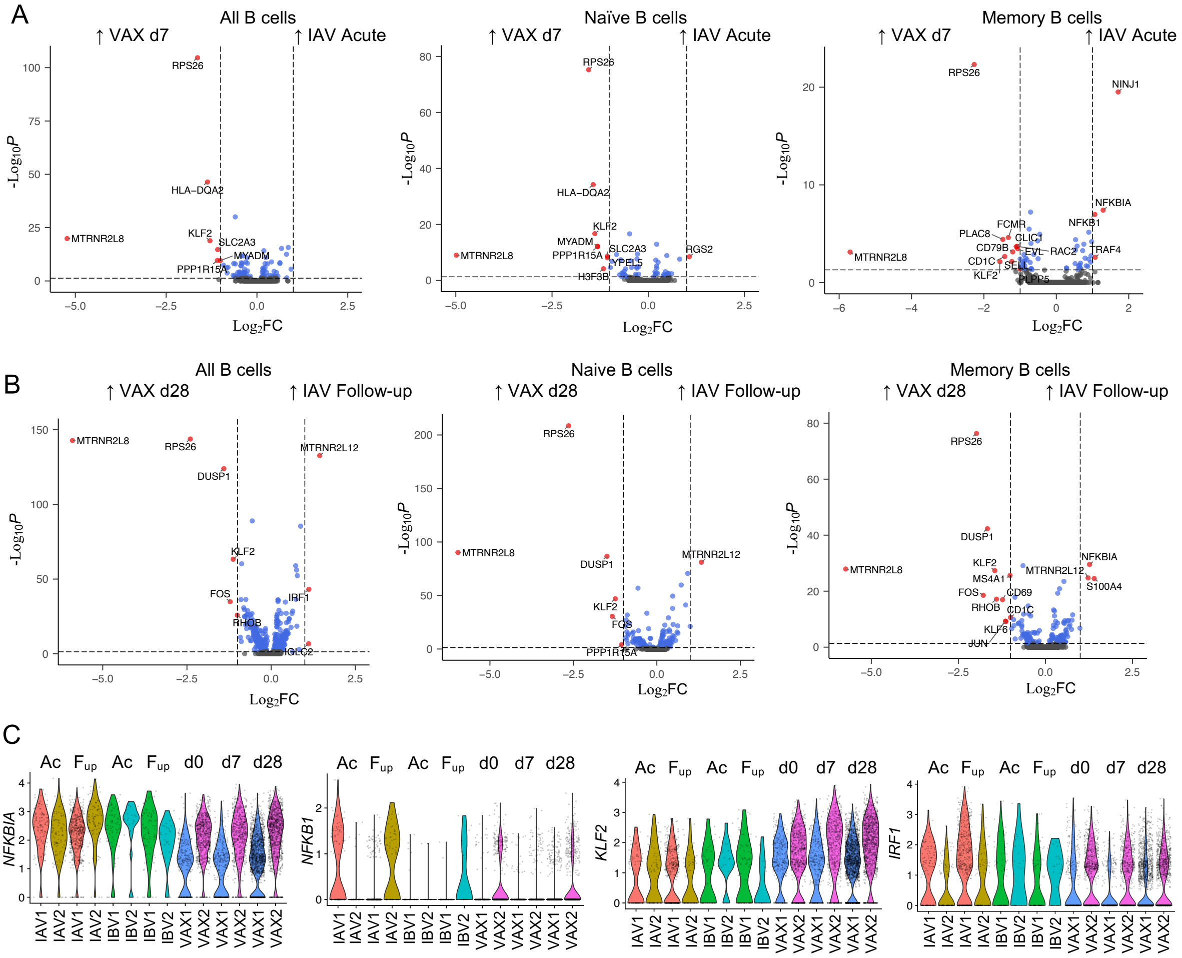

Appendix Figure S7. DEGs between vaccination and IAV infection. Volcano plots showing fold-changes of DEGs of total, naïve (clusters 0, 1, 6, 7, 8) and memory (clusters 2, 3_0, 4, 5, 9, 12) B cells for (**A**) IAV Acute vs VAX d7 and (**B**) IAV Follow-up vs VAX d28. (**C**) Violin plots of *NFKBIA*, *NFKB1, KLF2* and *IRF1*.

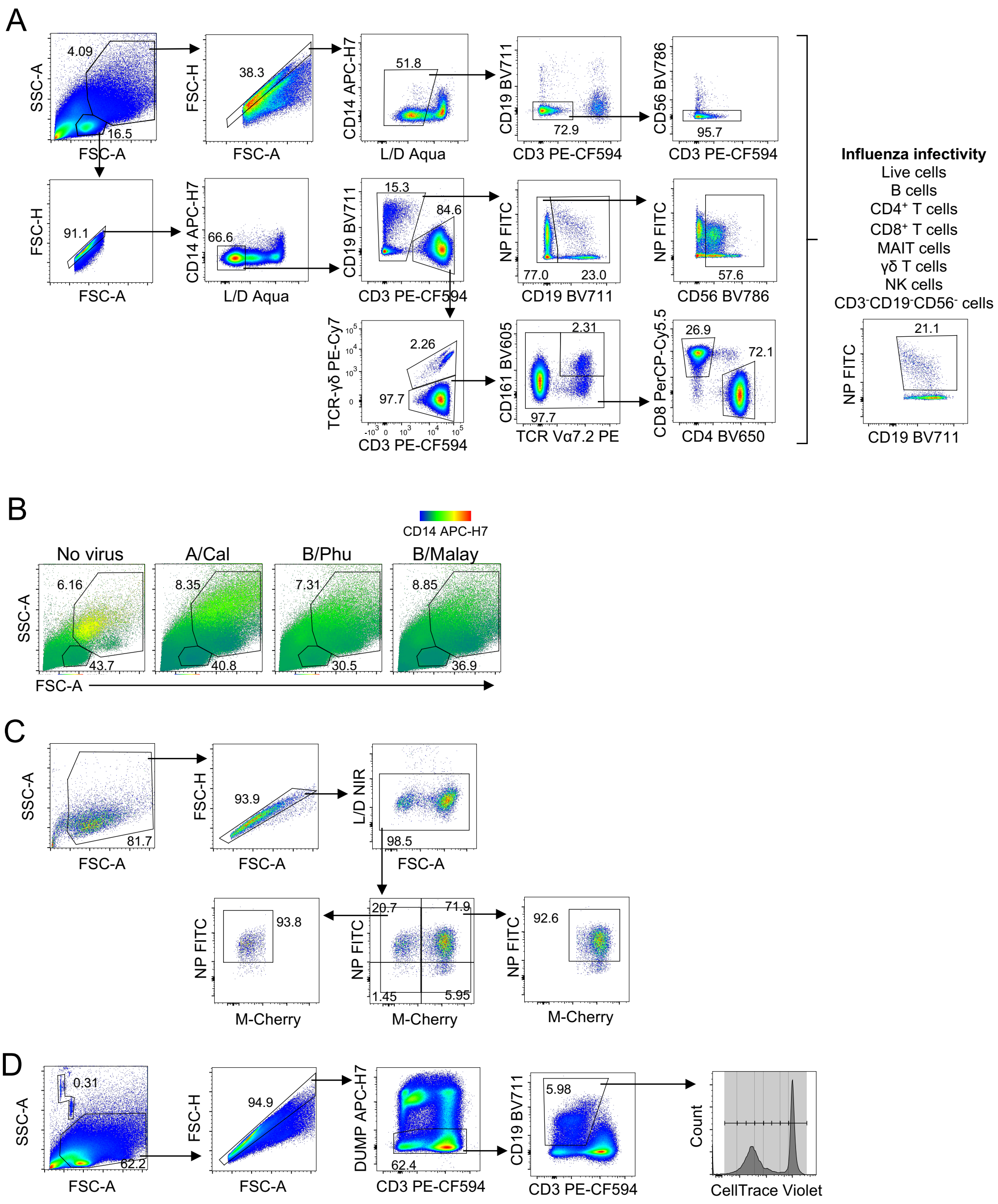

Appendix Figure S8. Flow cytometry gating strategies. Representative gating strategies for (**A**) influenza virus infection assay, (**B**) monocyte gating based on FSC-A and SSC-A, (**C**) A549 cell infection assay and (**D**) B cell proliferation assay.

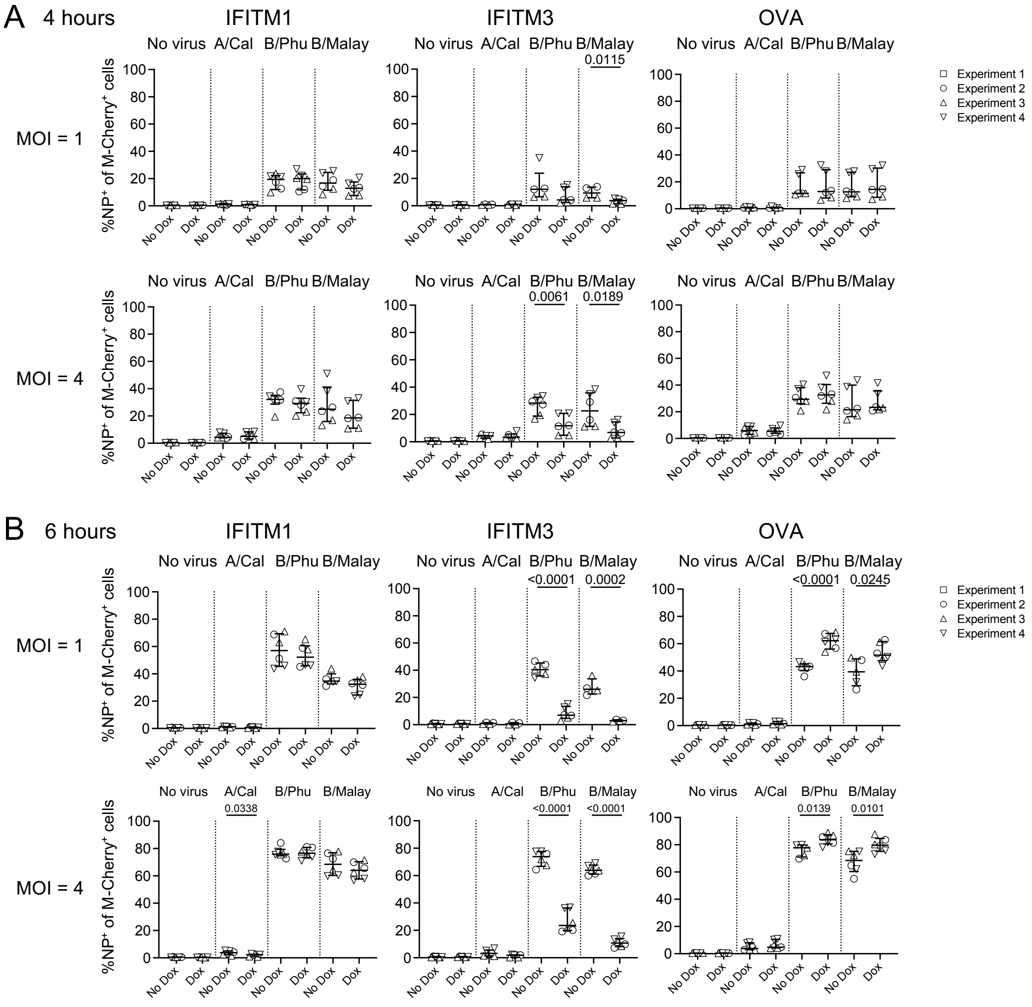

Appendix Figure S9. Influenza virus infectivity of A549 cell lines. (**A-B**) Frequency of NP^{+} A549 cells with inducible level of IFITM1, IFITM3 or OVA at (**A**) 4 hours and (**B**) 6 hours post-infection from 3-4 independent experiments. Bars indicate the median with IQR. Statistical significance was determined with a two-tailed paired t test.
