# Supplementary material for: IFN-gene signatures in B cells following influenza A and B virus infection and influenza vaccination
Source: EMBO Mol Med. 2026 Mar 9;18(4):1456–77. doi: 10.1038/s44321-026-00395-8 (PMC13083893; doi:10.1038/s44321-026-00395-8)
Supplement: Supplementary file 2 — Table EV1 [file 44321_2026_395_MOESM2_ESM.docx]

**Table EV1.** Demographical and immunological data of participants included in single-cell RNA sequencing.

| Study cohort | Infection time point | IAV1 (A/H1N1) | IAV2 (A/H1N1) | IBV1 (B/Yamagata) | IBV2 (B/Yamagata) | Vaccination time point | VAX1 (2015 Fluvax) | VAX2 (2016 Fluvax) |
| --- | --- | --- | --- | --- | --- | --- | --- | --- |
| Age (years) |  | 63 | 35 | 21 | 39 |  | 27 | 25 |
| Gender |  | Female | Female | Female | Female |  | Female | Female |
| HAI titre of the infection/vaccine strains: |  |  |  |  |  |  |  |  |
| A/H1N1: | Acute | 1280 | 80 | 5 | 5 | d0 | 10 | 40 |
|  | Follow-up | 2560 | 320 | 5 | 5 | d28 | 160 | 320 |
| A/H3N2: | Acute | 5 | 10 | 10 | 40 | d0 | 20 | 80 |
|  | Follow-up | 20 | 10 | 10 | 40 | d28 | 40 | 1280 |
| B/Yamagata: | Acute | 160 | 40 | 5 | 80 | d0 | 10 | 5 |
|  | Follow-up | 80 | 40 | 20 | 320 | d28 | 320 | 40 |
| B/Victoria: | Acute | 20 | 5 | 80 | 5 | d0 | – | 10 |
|  | Follow-up | 20 | 5 | 80 | 5 | d28 | – | 1280 |
| % ASC (CD27^+^CD38^+^) of B cells: |  |  |  |  |  | d0 | 3.72 | 0.29 |
|  | Acute | 11.04 | 3.68 | 12.48 | 2.35 | d7 | 6.2 | 7.04 |
|  | Follow-up | 2.71 | 0 | 0 | 1.08 | d28 | 1.21 | 0.36 |
| # ASC (CD27^+^CD38^+^): |  |  |  |  |  | d0 | 7.23 | 0.8 |
|  | Acute | 81.5 | 8.93 | 40.54 | 5.24 | d7 | 16.84 | 28.26 |
|  | Follow-up | 7 | 0 | 0 | 3.06 | d28 | 3.62 | 0.77 |
| % PD-1^+^ICOS^+^ Tfh1 (CXCR3^+^CCR6^-^) of CD4^+^ T cells: |  |  |  |  |  | d0 | – | 0.17 |
|  | Acute | 0.57 | 0.58 | 0.62 | 0.34 | d7 | 1.18 | 2.4 |
|  | Follow-up | 0.11 | 0 | 0.00103 | 0.16 | d28 | 0.38 | 0.26 |
| # PD-1^+^ICOS^+^ Tfh1 (CXCR3^+^CCR6^-^): |  |  |  |  |  | d0 | – | 1.75 |
|  | Acute | 4.47 | 7.11 | 6.31 | 9.55 | d7 | 12.87 | 28.18 |
|  | Follow-up | 1.84 | 0 | 0 | 3.55 | d28 | 4.52 | 1.98 |
| % A/Cal HA-specific of B cells: |  |  |  |  |  | d0 | 0.084 | 0.049 |
|  | Acute | 1.05 | 0.18 | – | – | d7 | 0.36 | 0.14 |
|  | Follow-up | 0.49 | 0.21 | – | – | d28 | 0.78 | 0.5 |
| # A/Cal HA-specific B cells: |  |  |  |  |  | d0 | 27.19 | 10.45 |
|  | Acute | 1402.12 | 72.8 | – | – | d7 | 203.69 | 48.16 |
|  | Follow-up | 284.55 | 125.64 | – | – | d28 | 418.76 | 108.04 |
| % B/Phu HA-specific of B cells: |  |  |  |  |  | d0 | 0.06 | 0.022 |
|  | Acute | – | – | – | – | d7 | 0.49 | 0.2 |
|  | Follow-up | – | – | – | – | d28 | 0.83 | 1.4 |
| # B/Phu HA-specific B cells: |  |  |  |  |  | d0 | 19.42 | 4.69 |
|  | Acute | – | – | – | – | d7 | 271.59 | 72.24 |
|  | Follow-up | – | – | – | – | d28 | 448.68 | 305.32 |
| Ethnicity |  | Caucasian | Caucasian | Caucasian | Non Aboriginal TSI |  |  |  |
| Days of ILI before hospitalization |  | 2 | 3 | 14 | 4 |  |  |  |
| Days in hospital |  | 4 | 6 | 7 | 2 |  |  |  |
| Admitted to |  | Respiratory/ general | Respiratory/ general | Respiratory/ general | Respiratory/ general |  |  |  |
| Clinical presentation |  | ILI | ILI, pneumonia | ILI | ILI |  |  |  |
| Oseltamivir |  | No | Yes | Yes | Yes |  |  |  |
| Days of oseltamivir treatment |  | NA | 6 | 2 | 10 |  |  |  |
| Oxygen support |  | None | Non-invasive | None | None |  |  |  |
| 30-day status |  | Alive | Alive | Alive | Alive |  |  |  |
| Weight (kg) |  | 95 | 92 | 64 | 57.4 |  |  |  |
| Height (cm) |  | 167 | 157 | 170 | 158 |  |  |  |
| BMI |  | 34.1 | 37.3 | 22.1 | 23.0 |  |  |  |
| Chronic respiratory disease |  | Yes | No | No | Yes |  |  |  |
| Cardiac disease |  | No | No | No | Yes |  |  |  |
| Chronic renal disease |  | No | No | No | Yes |  |  |  |
| Immunosuppressants |  | Yes (hydrocortisone) | None | None | Yes |  |  |  |
| Smoker |  | Current | Past | Current | No |  |  |  |
| Hospital admission in the past 12 months |  | No | No | No | Yes |  |  |  |
| Vaccination within the infected year |  | No | No | No | Yes |  |  |  |
| Prior vaccination (last 2 years) |  | No | No | No | No |  |  |  |

Abbreviations: ILI, influenza-like illness.
